# Editorial: Frontiers in Autoimmune Disease: Rheumatic Fever and Rheumatic Heart Disease

**DOI:** 10.3389/fped.2015.00091

**Published:** 2015-10-28

**Authors:** Luiza Guilherme, Karen F. Köhler, Kellen C. Faé

**Affiliations:** ^1^Laboratory of Immunology, Heart Institute (InCor), School of Medicine, University of São Paulo, São Paulo, Brazil

**Keywords:** rheumatic fever, rheumatic heart disease, autoimmune diseases, valvular damage, innate and adaptive immune response, animal model

Rheumatic fever (RF) and rheumatic heart disease (RHD), its most clinically consequence, resulting from untreated throat *Streptococcus pyogenes* infection in susceptible children, are considered as models of autoimmune post-infectious disease. This Research Topic compiled clinical and scientific data and brings interesting viewpoints of clinicians and basic researchers. The ensemble of data and ideas certainly makes a new portrait of the mechanisms leading the autoimmune reactions. On the other hand, the clinical data contributes with the diagnosis and prevention of the disease.

Rheumatic fever and RHD are still prevalent in diverse regions of the world (1–3). The correct diagnosis is very important as well as the clinical treatment.

Briefly, in Brazil, both RF and RHD are still important diseases in different regions (4). A RF prevention program involving more than 700 children with RF/RHD was performed. This program evaluated the long-term evolution and outcomes after the control of recurrences. These data are presented and discussed by Mota et al. (5). Another interesting article, done by Spina et al., discusses the clinical diagnosis of acute rheumatic myocarditis in asymptomatic RHD patients (6).

As RF and RHD are autoimmune diseases, the mechanisms leading to autoimmune reactions involve several molecules that play a role in the immune response against the bacteria (7, 8). As we know, protective response involves several molecules that are genetically controlled, from both innate and adaptive immune response, in order to eradicate an infection (8). Among these molecules, complement plays an important role in the immune response against *S. pyogenes*. The lectin pathway of complement and RHD is discussed by the group de Messias-Reason (9).

Another interesting article on autoimmune targets is presented by Root-Bernstein. In his article, a parallel between molecular mimicry reactions on RHD and autoimmune myocarditis is established (10).

Rheumatic fever and RHD are considered as prototypes of human autoimmune diseases, and no animal models could reproduce accurately the disease. In the past few years, however, experiments on Lewis rat showed some similar autoimmune reactions in the myocardium of *S. pyogenes* immunized mice. The article by the group of Ketheesan presented a historical overview of animal models that were used to investigate the pathogenesis of RF/RHD (11).

I hope the readers enjoy this collection, and I and my co-editors are grateful of having the opportunity to prepare this Research Topic for Frontiers in Pediatrics.

## Conflict of Interest Statement

The authors declare that the research was conducted in the absence of any commercial or financial relationships that could be construed as a potential conflict of interest.

## References

[1] RHD Australia (ARF/RHD Writing Group), National Heart Foundation of Australia and the Cardiac Society of Australia and New Zealand. Australian Guideline for Prevention, Diagnosis and Management of Acute Rheumatic Fever and Rheumatic Heart Disease (2012).

[2] Marijon E Ou P Celermajer DS Ferreira B Mocumbi AO Jani D Prevalence of rheumatic heart disease detected by echocardiographic screening. N Engl J Med (2007) 357:470–6. 10.1056/NEJMoa065085 17671255

[3] Marijon E Celermajer DS Tafflet M El-Haou S Jani DN Ferreira B Rheumatic heart disease screening by echocardiography: the inadequacy of World Health Organization criteria for optimizing the diagnosis of subclinical disease. Circulation (2009) 120:663–8. 10.1161/CIRCULATIONAHA.109.849190 19667239

[4] Barbosa PJB Müller RE Diretrizes Brasileiras para o Diagnóstico, Tratamento e Prevenção da Febre Reumática Arquivo Brasileiro de Cardiologia. Arq Bras Cardiol (2009) 93 (3 supl 4):1–18. 20976376

[5] Mota CC Meira ZM Graciano RN Graciano FF Araújo FD. Rheumatic fever prevention program: long-term evolution and outcomes. Front Pediatr (2015) 2:141. 10.3389/fped.2014.00141 25610826 PMC4285057

[6] Spina GS Sampaio RO Branco CE Miranda GB Rosa VEE Tarasoutchi F. Incidental histological diagnosis of acute rheumatic myocarditis: case report and review of the literature. Front Pediatr (2014) 2:126. 10.3389/fped.2014.00126 25478552 PMC4238348

[7] Cunningham MW. Pathogenesis of group A streptococcal infections. Clin Microbiol Rev (2000) 13:470–511. 10.1128/CMR.13.3.470-511.2000 10885988 PMC88944

[8] Guilherme L Kalil J. Rheumatic heart disease: molecules involved in valve tissue inflammation leading to the autoimmune process and anti-*S. pyogenes* vaccine. Front Immunol (2013) 4:352. 10.3389/fimmu.2013.00325 24198818 PMC3812567

[9] Beltrame MH Catarino SJ Goeldner I Boldt ABW de Messias-Reason IJ. The lectin pathway of complement and rheumatic heart disease. Front Pediatr (2015) 2:148. 10.3389/fped.2014.00148 25654073 PMC4300866

[10] Root-Bernstein R. Rethinking molecular mimicry in rheumatic heart disease and autoimmune myocarditis: laminin, collagen IV, CAR, and B1AR as initial targets of disease. Front Pediatr (2014) 2:85. 10.3389/fped.2014.00085 25191648 PMC4137453

[11] Rush CM Govan BL Sikder S Williams NL Ketheesan N. Animal models to investigate the pathogenesis of rheumatic heart disease. Front Pediatr (2014) 2:116. 10.3389/fped.2014.00116 25414841 PMC4220098

